# Cardiac safety profiles of first-generation vs second-generation BTK inhibitors: a meta-analysis

**DOI:** 10.1093/oncolo/oyag102

**Published:** 2026-03-23

**Authors:** Ali Mushtaq, Eman Nayaz Ahmed, Roaa Aljumaa, Abdel Rahman E’mar, Osamah Badwan, Omer Ashruf, Ahmad Elshaer, Abdullah Shaik, Mohanad Baroudi, Rohit Moudgil, Moaath K Mustafa Ali

**Affiliations:** Department of Internal Medicine, Cleveland Clinic, Cleveland, OH, 44195, United States; College of Medicine, Alfaisal University, Riyadh, 11533, Saudi Arabia; College of Medicine, Alfaisal University, Riyadh, 11533, Saudi Arabia; Department of Cardiology, Cleveland Clinic, Cleveland, OH, 44195, United States; Department of Cardiology, Cleveland Clinic, Cleveland, OH, 44195, United States; Indiana University School of Medicine Department of Internal Medicine, Indianapolis, IN, 46202, United States; Department of Medicine, Creighton University School of Medicine, Omaha, NE, 68178, United States; Department of Internal Medicine Henry Ford St. John Hospital, Detroit, MI, 48236, United States; Department of Internal Medicine, Cleveland Clinic, Cleveland, OH, 44195, United States; Department of Cardiology, Cleveland Clinic, Cleveland, OH, 44195, United States; Cleveland Clinic Taussig Cancer Center, Cleveland, OH, 44106, United States

**Keywords:** Bruton’s tyrosine kinase inhibitors, B-cell malignancy, atrial fibrillation, cardiovascular outcomes, cardiac safety profile, ibrutinib, acalabrutinib, zanubrutinib, meta-analysis, cardiotoxicity

## Abstract

**Background:**

The first-generation Bruton’s tyrosine kinase inhibitor (BTKi), ibrutinib, is associated with significant cardiotoxicity. Second-generation agents were developed to mitigate this risk and offer an improved safety profile. This systematic review and meta-analysis of six direct comparator studies compares the cardiac safety profiles of first- and second-generation BTKi.

**Methods:**

We systematically searched Medline, Embase, and Cochrane for studies directly comparing first- and second-generation BTKi. Data from 6 studies, encompassing 14 455 patients (12 816 in the first-generation arm and 1639 in the second-generation arm), were included. The primary outcomes were the incidence of atrial fibrillation (AF) and cardiac events, defined by the Medical Dictionary for Regulatory Activities system organ class. All pooled analyses were conducted using a random-effects model. Publication bias was evaluated visually using a funnel plot, quantitatively with Egger’s regression test, and corrected using the Duval and Tweedie trim-and-fill method.

**Results:**

Compared to second-generation agents, ibrutinib was associated with a significantly higher risk of AF (OR 2.50, 95% CI, 1.97-3.17), total cardiac events (OR 1.53, 95% CI, 1.18-1.99), and heart failure (OR 2.08, 95% CI, 1.13-3.83). This translated to a 3-fold higher rate of treatment discontinuation for a cardiac cause (OR 3.32, 95% CI, 1.46-7.55). No significant difference in all-cause mortality was found.

**Conclusion:**

Second-generation BTKi may provide a more favorable cardiovascular safety profile than ibrutinib, resulting in fewer key cardiac events and less treatment-limiting toxicity. These findings should inform clinical decision-making, especially for patients with increased risk for cardiovascular disease.

## Graphical Abstract

**Graphical Abstract:**
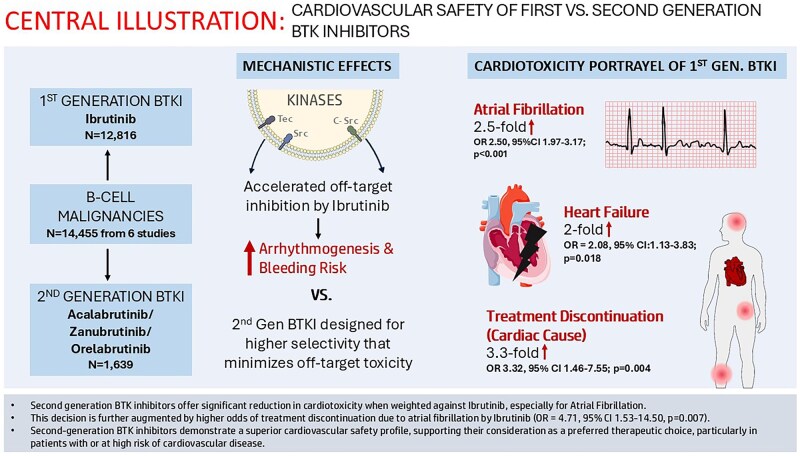

Implications for Practice This meta-analysis of 14 455 patients across six direct comparator studies demonstrates that first-generation ibrutinib is associated with a significantly higher risk of atrial fibrillation (OR 2.50), total cardiac events (OR 1.53), and heart failure (OR 2.08) compared to second-generation BTK inhibitors. Critically, the increased cardiotoxicity is not merely an incidental finding, as patients receiving ibrutinib faced a more than 3-fold higher rate of treatment discontinuation due to a cardiac cause (OR 3.32), directly compromising cancer therapy continuity. These findings support the upfront selection of a second-generation BTK inhibitor in patients with pre-existing cardiovascular disease, a history of atrial fibrillation, or multiple cardiac risk factors, where the treatment-limiting toxicity burden of ibrutinib is likely to be greatest.

## Introduction

Bruton’s tyrosine kinase inhibitors (BTKi) have transformed the therapeutic landscape for B-cell malignancies, including chronic lymphocytic leukemia, Waldenström macroglobulinemia, and mantle cell lymphoma.^1–10^ These agents function by irreversibly binding to the BTK enzyme, thereby inhibiting B-cell receptor signaling, which is critical for the proliferation and survival of malignant B-cells.^11^

Ibrutinib, the first-in-class BTKi approved in 2013, produced durable responses across multiple B-cell cancers^12^ but was subsequently linked to a concerning rate of cardiovascular adverse events, primarily atrial fibrillation (AF), hypertension, and bleeding.^2^^,^^5–9^ With AF rates reported as high as 20% in early studies, alongside incidence rates reaching up to 29% for hypertension and 7% for heart failure, the long-term cardiovascular safety of ibrutinib became a significant clinical concern, particularly in older patients with comorbidities.^13–16^ This elevated risk was consistently confirmed in subsequent meta-analyses, underscoring the urgent need for safer therapeutic alternatives,^13^^,^^17^ especially in this high-risk patient population.

Selective second-generation BTKi have received approval from the Food and Drug Administration: Acalabrutinib was approved in 2017, Zanubrutinib was approved in 2019, and the more recent addition is Orelabrutinib.^3^^,^^18^^,^^19^ These agents were engineered to minimize off-target kinase inhibition, a key mechanism thought to be implicated in ibrutinib’s cardiotoxicity, thereby potentially offering an improved safety profile.^20^^,^^21^ While individual head-to-head trials have suggested lower rates of cardiac events with these newer agents, uncertainty remains regarding the overall magnitude and consistency of this safety benefit across the entire class.

Accordingly, this systematic review and meta-analysis aim to consolidate the data from all direct comparator studies to definitively quantify the relative cardiovascular risks of first- versus second-generation BTKi. Our findings are intended to guide clinical decision-making, particularly for patients at an elevated risk of cardiac disease.

## Materials and methods

### Search strategy and study selection

This systematic review and meta-analysis were conducted and reported in accordance with the Preferred Reporting Items for Systematic Reviews and Meta-Analyses (PRISMA) guidelines. A systematic and comprehensive search of Medline (via Ovid SP), Embase (via Ovid SP), and the Cochrane Central Register of Controlled Trials (CENTRAL) was performed to identify all relevant studies published up to May 28, 2024. The search strategy combined Medical Subject Headings and free-text terms for Bruton’s tyrosine kinase inhibitors and cardiovascular events.

The core search logic included terms such as: (ibrutinib OR first-generation BTK inhibitor) AND (acalabrutinib OR zanubrutinib OR orelabrutinib OR second-generation BTK inhibitor) AND (cardiac OR cardiotoxicity OR myocardial infarction OR atrial fibrillation OR arrhythmia OR heart failure OR hypertension). While the search string was optimized for primary cardiac events, bleeding outcomes were universally extracted from the eligible comparative literature. The reference lists of included studies and relevant review articles were also manually scanned to identify any additional eligible reports. Both controlled vocabulary and free-text terms were utilized. Articles were imported into EndNote and then transferred to Covidence, where the screening process was conducted.^22^

### Eligibility criteria and study selection

Studies were included if they met the following criteria:

- Study Design: Randomized controlled trials or observational (prospective or retrospective cohort) studies.
- Population: Patients with any B-cell malignancy.
- Intervention: Treatment with a second-generation BTKi (acalabrutinib, zanubrutinib, or orelabrutinib).
- Comparator: Direct comparison with the first-generation BTKi (ibrutinib).
- Outcomes: Reported data on the incidence of at least one predefined cardiovascular adverse event in both treatment arms.

Studies were excluded if they were single-arm non-comparative trials, case reports, reviews, meta-analysis, editorials, or conference abstracts.

**Figure 1.**
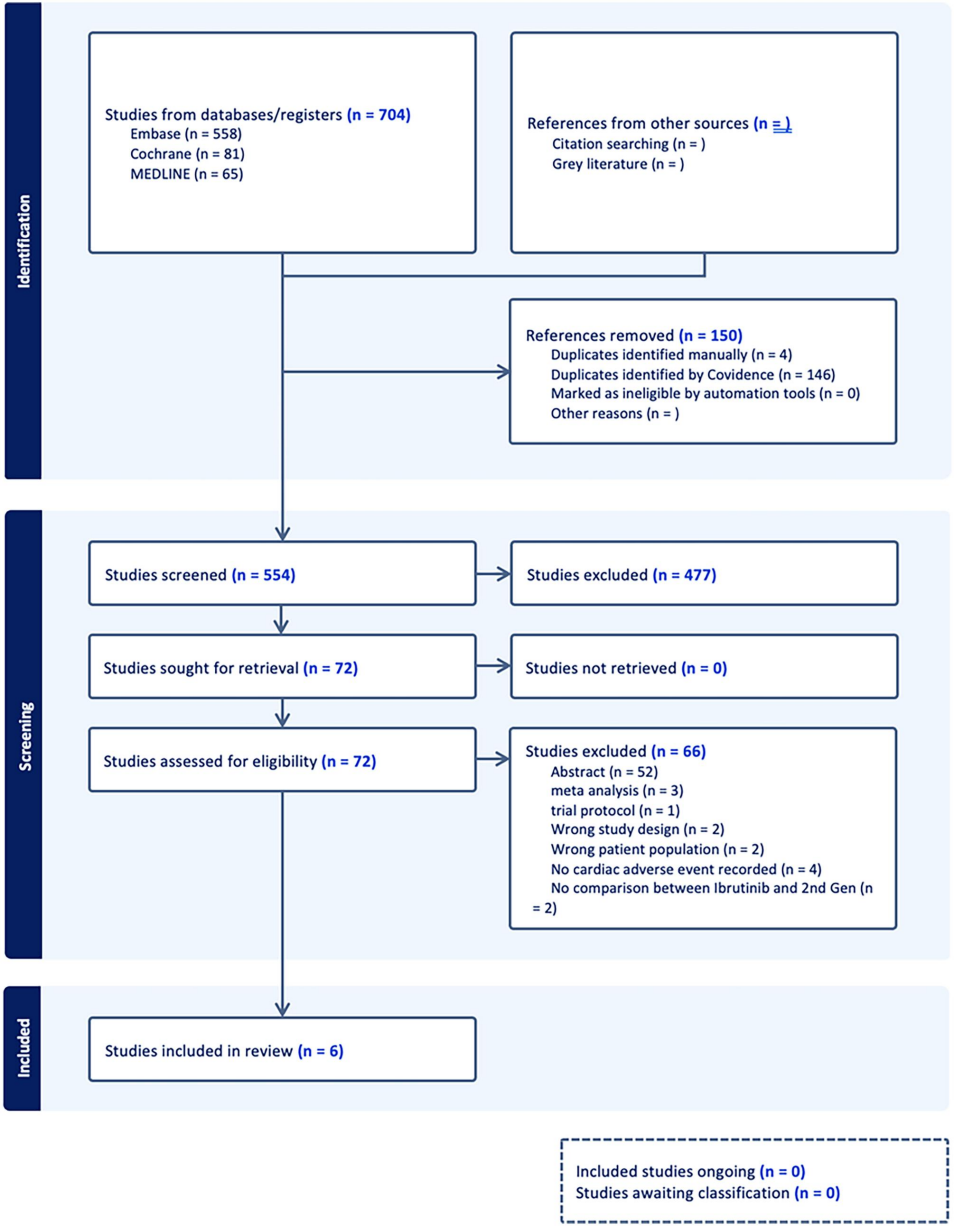
PRISMA Flow diagram

Two investigators (R.A. and E.N.A.) independently screened titles and abstracts using Covidence screening software.^22^ The full texts of potentially eligible articles were then retrieved and assessed against the eligibility criteria. Any disagreements during the screening or selection process were resolved by consensus, with consultation of a third reviewer (A.M.) if consensus was not reached. Discrepancies at the full-text screening stage, as well as reviewing all the final included studies, were resolved by the third independent reviewer (A.M.). Data from the included studies were independently extracted by two reviewers using a standardized data collection form. The extracted information included: 1. Study Characteristics: First author, publication year, study design, and follow-up duration. 2. Patient Demographics: Sample size, age, sex, and type of B-cell malignancy. 3. Data for all outcomes were extracted as dichotomous event counts. For each predefined outcome, we collected the total number of patients experiencing the event and the total number of patients in the corresponding treatment arm. We prioritized this format to calculate odds ratios, as event-count data was the most consistently reported format across both randomized trials and observational studies, allowing for a comprehensive synthesis of all available evidence. Figure 1 details the PRISMA diagram.

### Risk of bias assessment

The methodological quality of the included studies was independently assessed by two reviewers. The Cochrane Risk of Bias 2 tool was utilized for randomized controlled trials, evaluating bias arising from the randomization process, deviations from intended interventions, missing outcome data, measurement of the outcome, and selection of the reported result.^23^ The Newcastle-Ottawa Scale was used for observational studies to assess the risk of bias based on cohort selection, comparability of the groups, and ascertainment of the outcome.^24^

### Outcome of interest

The primary outcomes were the incidence of atrial fibrillation (AF) and total cardiac events. Total cardiac events was a composite outcome defined as any adverse event captured under the Medical Dictionary for Regulatory Activities’ Cardiac System Organ Class. This classification includes, but is not limited to, cardiac arrhythmias, coronary artery disorders, heart failure, and myocardial disorders.^25^ Secondary outcomes included individual incidence of heart failure, hypertension, coronary artery disease (defined as a composite including acute coronary syndromes and events related to chronic ischemic heart disease), bleeding events (as defined by each individual included study), ventricular tachycardia, all-cause mortality, treatment discontinuation due to AF, and treatment discontinuation due to any cardiac event.

### Statistical analysis

**Figure 2.**
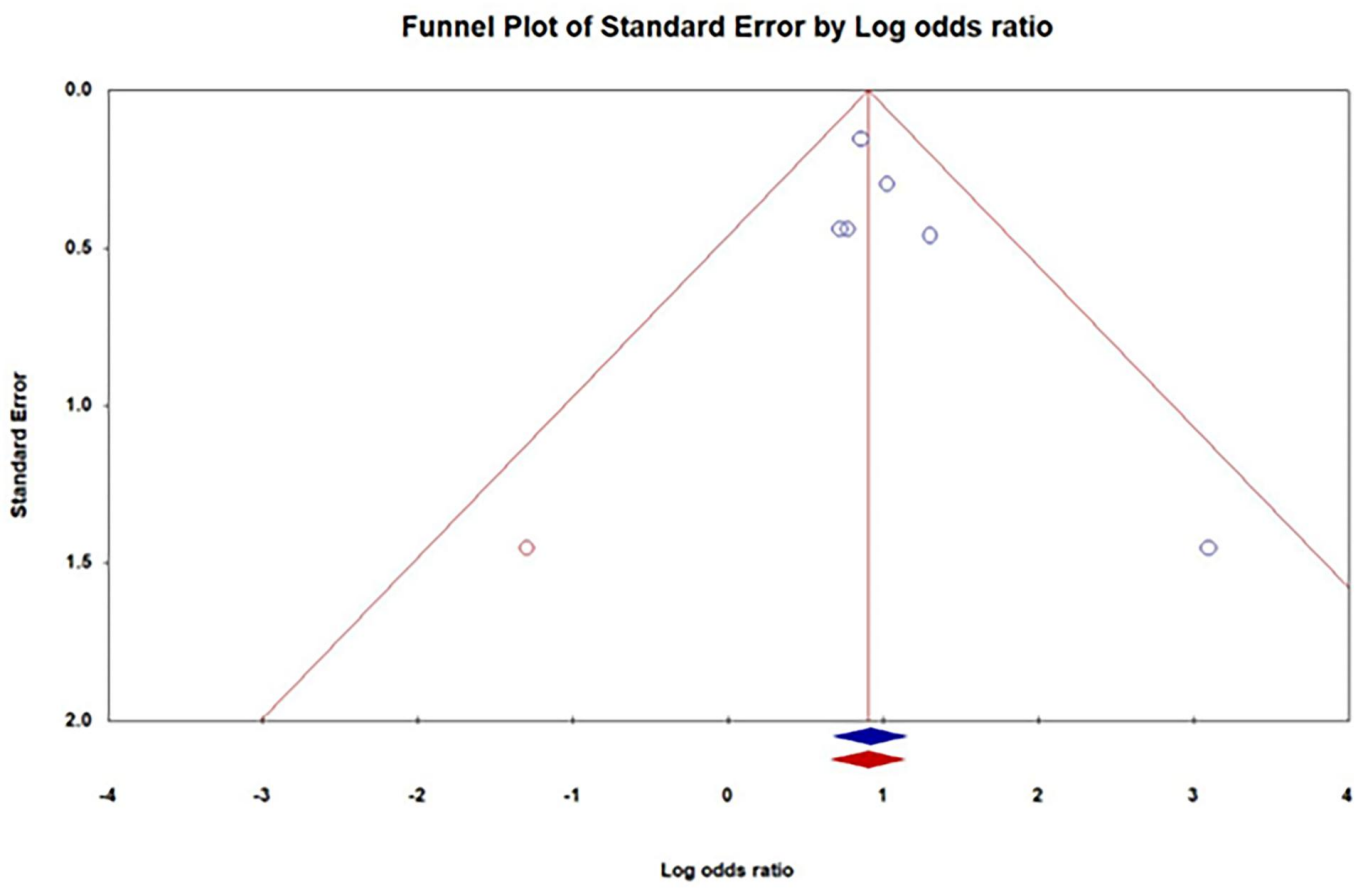
Funnel Plot of Atrial Fibrillation Risk

All statistical analyses were conducted using Comprehensive Meta-Analysis software, Version 3.3 (Biostat, Englewood, NJ).^26^ The effect measure for all dichotomous outcomes was the odds ratio (OR) with its corresponding 95% confidence interval (CI). Given the clinical and methodological diversity of the included studies (eg, different trial designs and patient populations), a random-effects model (DerSimonian and Laird) was used for all pooled analyses.^27^ Statistical heterogeneity among studies was quantified using the *I*^2^ statistic, and interpreted as low (<25%), moderate (25%-50%), or substantial (>50%). Potential publication bias for the primary outcome of AF was assessed by visual inspection of a funnel plot of the effect estimates against their standard errors. Egger’s regression test was used to statistically test for plot asymmetry. In the case of suspected bias, the Duval and Tweedie trim-and-fill method was employed to estimate an adjusted OR.^28^^,^^29^ A funnel plot is shown in Figure 2. A two-tailed *P*-value of ≤.05 was considered statistically significant for all analyses.

## Results

### Study selection and characteristics

The PRISMA search and screening process is detailed in Figure 1. A total of 6 studies met the inclusion criteria and were included in the meta-analysis. These studies comprised a total of 14°455 patients, with 12°816 allocated to the first-generation BTKi (ibrutinib) arm and 1639 to a second-generation BTKi arm. The included studies consisted of three randomized controlled trials and three retrospective/observational cohort studies.

### Risk of bias assessment

The three randomized controlled trials (ELEVATE-RR, ALPINE, ASPEN) were evaluated using the Cochrane RoB 2 tool.^30–32^ Two trials were judged to have a low overall risk of bias, while one was judged to have “some concerns” due to its open-label design, though the risk of bias for objective outcomes like mortality and AF was still considered low. The three observational studies were assessed using the Newcastle-Ottawa Scale. The retrospective cohort studies by Roeker et al. and Qiao et al. were deemed to be of good quality (scoring 8 and 7 out of 9 stars, respectively).^33^^,^^34^ The pharmacovigilance study by Zhai et al. was rated as having a high risk of bias (4 stars) due to the inherent limitations of spontaneous adverse event reporting systems.^35^ Overall, the methodological quality of the included studies was judged to be high, primarily comprising evidence from randomized trials and good-quality observational studies (Table S1).

### Primary outcomes

**Figure 3.**
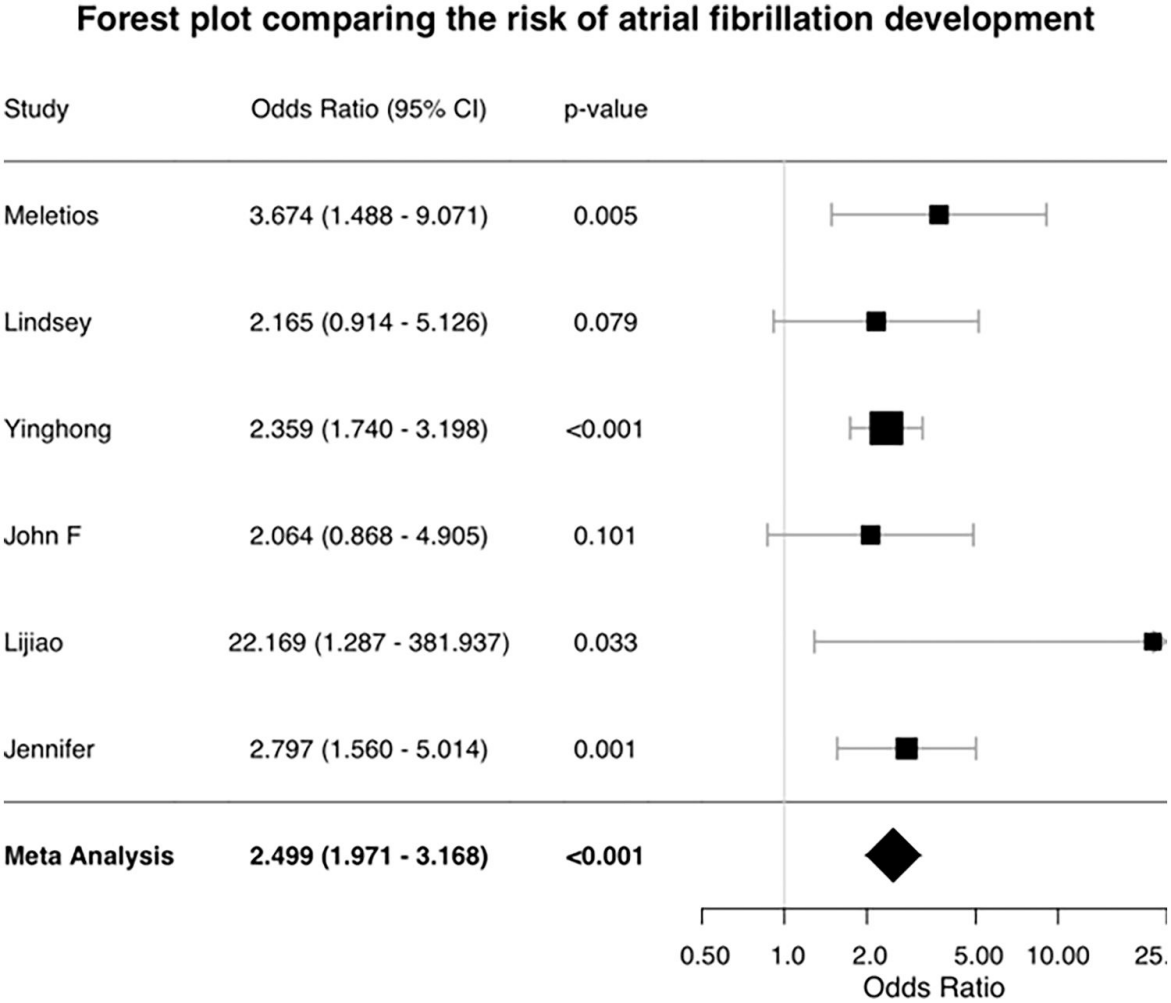
Forest plot comparing the risk of atrial fibrillation between first- and second- generation BTK inhibtors

A summary of all pooled analyses is presented in Table 1. The pooled analysis demonstrated a significantly lower risk of cardiotoxicity with second-generation agents. The odds of AF were significantly higher with ibrutinib, with the pooled analysis showing a 2.5-fold increase in risk (OR 2.50, 95% CI, 1.97-3.17; *P* < .001), with no significant heterogeneity (*I*^2^ = 0%). A forest plot for this outcome is shown in Figure 3. Similarly, the odds of total cardiac events were increased by 53% with ibrutinib (OR 1.53, 95% CI, 1.18-1.99; *P* = .001) compared to second-generation BTKi (Figure S1).

**Table 1.** Summary of meta-analysis results for cardiovascular outcomes.

Outcome	No. of studies	Odds ratio (OR)	95% confidence interval (CI)	*P*-value	Heterogeneity (*I*^2^)	Conclusion
Atrial fibrillation	6	2.5	1.97-3.17	<.001	0%	Significantly higher risk with ibrutinib
General cardiac events	3	1.53	1.18-1.99	.001	0%	Significantly higher risk with ibrutinib
Heart failure	4	2.08	1.13-3.83	.018	53.70%	Significantly higher risk with ibrutinib
Treatment Discontinuation (any cardiac cause)	3	3.32	1.46-7.55	.004	0%	Significantly higher risk with ibrutinib
Treatment discontinuation (atrial fibrillation)	3	4.71	1.53-14.50	.007	0%	Significantly higher risk with ibrutinib
Hypertension	3	3.88	.96-15.78	.058	32.30%	Trend towards higher risk with ibrutinib
Bleeding events (any)	5	1.67	.90-3.09	.105	73.70%	Trend towards higher risk with ibrutinib
Coronary artery disease	3	1.1	.77-1.56	.599	0%	No significant difference
Ventricular tachycardia	4	1.97	.74-5.22	.175	8.10%	No significant difference
Mortality	5	1.13	.77-1.67	.521	0%	No significant difference

### Secondary outcomes

For specific cardiac outcomes, ibrutinib was associated with a 2-fold higher odds of heart failure (OR = 2.08, 95% CI, 1.13-3.83; *P* = .018) with moderate heterogeneity noted (*I*^2^ = 53.7%) (Figure S2). For hypertension, the point estimate suggested a nearly 4-fold increase in odds with ibrutinib (OR 3.88); however, the confidence interval was wide and included the null value, indicating imprecision in this estimate (95% CI, 0.96-15.78; *P* = .058) (Figure S3). No significant difference was observed for coronary artery disease (OR 1.10, 95% CI, 0.77-1.56; *P* = .599). Notably, although ibrutinib was associated with a numerically higher odds of ventricular tachycardia compared to second-generation agents, this difference did not reach statistical significance (OR 1.97, 95% CI 0.74-5.22; *P* = .175), likely reflecting limited statistical power due to the low incidence of this outcome across included studies. These results are detailed in Table 1 and Figure S4.

#### Bleeding and mortality

Ibrutinib was associated with a trend toward a higher risk of major bleeding events (OR = 1.67, 95% CI, 0.90-3.09; *P* = .105) (Figure S5), with substantial heterogeneity observed (*I*^2^ = 73.7%). There was also no significant difference in all-cause mortality between the first- and second-generation BTKi (OR = 1.13, 95% CI, 0.77-1.67; *P* = .521).

#### Treatment discontinuation

Reflecting the clinical impact of these adverse events, treatment discontinuation due to cardiac toxicity was significantly more frequent with ibrutinib. The odds of discontinuing treatment due to any cardiac event were over 3 times higher in the ibrutinib arm (OR = 3.32, 95% CI, 1.46-7.55; *P* = .004) (Figure S6). Specifically focusing on arrhythmia, discontinuation due to AF was also significantly more common with ibrutinib (OR = 4.71, 95% CI, 1.53-14.50; *P* = .007) (Figure S7) than second-generation BTKi.

### Publication bias

For the primary outcome of AF, the funnel plot showed slight asymmetry. However, Egger’s regression test did not indicate statistically significant publication bias (*P* = 0.16). Furthermore, the trim-and-fill analysis imputed one potential missing study, yielding an adjusted OR of 2.46 (95% CI, 1.94-3.12), which confirms the robustness of the primary finding.

## Discussion

This systematic review and meta-analysis of 6 direct comparator studies provide a robust quantitative synthesis of the comparative cardiovascular risk between first- and second-generation BTKi. Ibrutinib was associated with a significantly higher odds of AF (OR 2.50), total cardiac events (OR 1.53), and heart failure (OR 2.08). Our study found a 4-fold increase in treatment discontinuation due to AF in the ibrutinib arm.

Our pooled estimate for AF (OR 2.50) is consistent with and reinforces the results of the pivotal randomized trials included in our analysis. The ELEVATE-RR trial reported a significantly lower incidence of any-grade AF/flutter with acalabrutinib versus ibrutinib (9.4% vs 16.0%).^36^ Similarly, the final report from the ALPINE study noted a greater than 50% relative reduction in AF incidence with zanubrutinib versus ibrutinib (7.1% vs 17.0%).^31^ Our analysis corroborates a consistent class effect favoring second-generation agents and underscores the clinical reality that ibrutinib’s cardiotoxicity remains a leading cause of treatment discontinuation. This increased discontinuation risk likely reflects ibrutinib’s broader kinase inhibition and off-target effects, which may contribute to its inferior cardiovascular safety profile.^30^^,^^32–41^

The difference in cardiovascular safety between BTKi generations stems from their distinct kinase selectivity. Ibrutinib’s cardiotoxicity is largely attributed to its off-target inhibition of C-terminal Src kinase in cardiomyocytes, which leads to arrhythmogenesis.^42^ Similarly, its off-target effects on Tec and Src kinases contribute to the higher bleeding risk.^43–45^ Second-generation agents were specifically engineered for greater selectivity to minimize these off-target effects, a molecular advantage that our pooled clinical data corroborates.^3^^,^^18^^,^^20^^,^^21^

Despite the elevated risk in cardiotoxicity observed in our pooled analysis and the included individual trials, there was no significant difference in all-cause mortality between BTKi generations. While the ELEVATE-RR trial demonstrated non-inferior progression-free survival (PFS) for acalabrutinib, the more recent ALPINE trial showed a definitive PFS advantage for zanubrutinib over ibrutinib, suggesting superior disease control may eventually translate to a survival benefit with longer follow-up.^30^^,^^31^ Further, most cardiac events, including AF, are clinically manageable and rarely fatal, but the morbidity and management burden (eg, need for anticoagulation) remain substantial. If therapy is discontinued due to toxicity, patients often transition successfully to alternative agents, possibly mitigating the impact on overall survival (OS). Additionally, the indolent course of CLL and the availability of multiple effective subsequent therapies further dampen differences in OS, even when PFS outcomes diverge.

Beyond the incidence of any single adverse event, one of the most critical downstream consequences of cardiotoxicity is its impact on sustaining treatment. Our finding of a 3-fold higher rate of treatment discontinuation due to cardiac events with ibrutinib is clinically significant. The premature cessation of a primary anticancer agent due to toxicity can compromise disease control and has been linked to poor long-term outcomes.^30^^,^^39^^,^^46^^,^^47^ For patients with pre-existing cardiovascular conditions, such as a history of AF or heart failure, or those with multiple risk factors, as commonly observed in older populations with B-cell malignancies, our findings strongly support the upfront use of a second-generation agent.^48^ This decision-making should be preceded by a thorough baseline cardiovascular risk assessment, including a detailed history, an electrocardiogram, and optimization of modifiable risk factors like hypertension and diabetes. This approach aligns with the recent cardio-oncology consensus statements, which emphasize baseline cardiovascular risk stratification and recommend favoring agents with lower toxicity profiles in high-risk individuals.^49^ A multidisciplinary cardio-oncology team approach is essential to optimize both cancer treatment and cardiovascular health in this population.^50^

This meta-analysis has notable strengths, including its large patient population and the inclusion of recent, high-quality, head-to-head randomized trials. By synthesizing results from multiple direct comparator studies, we provide a higher level of evidence than any single trial alone. However, limitations must be acknowledged. The inclusion of observational studies introduces a potential for confounding, and significant heterogeneity was noted for the outcomes of heart failure and bleeding, suggesting variability across studies. For example, definitions of bleeding varied across studies, which may have contributed to heterogeneity. Moreover, the statistical power to detect differences in rarer outcomes like ventricular tachycardia remains limited. Differences in cumulative drug exposure and follow-up duration between both generations could introduce bias, specifically with observational studies. In particular, since ibrutinib has been available since 2013, real world cohorts of ibrutinib treated patients reflect substantially longer treatment exposures than those receiving more recently approved second generation agents, which could in part result in higher absolute capture of adverse events over time. It is plausible that trial eligibility criteria for second-generation agents were refined over time to exclude patients with severe baseline cardiovascular risk, informed by early toxicity signals from ibrutinib. While this source of confounding is minimized in the head-to-head RCTs included in our analysis, comparisons involving historical ibrutinib cohorts may be susceptible to this bias, potentially amplifying the perceived safety advantage of newer agents. Finally, our analysis was focused solely on cardiovascular events and did not capture other toxicities, such as headache or cytopenia, which can also influence treatment selection. Similarly, we did not evaluate the impact of drug cost and accessibility, which are important real-world factors in clinical decision-making. Looking forward, long-term follow-up from the pivotal trials is essential to determine if the superior safety and PFS of second-generation agents will ultimately translate into an OS benefit.

In conclusion, this meta-analysis provides robust evidence that second-generation BTKi may confer a significant and clinically meaningful reduction in the risk of atrial fibrillation, heart failure, and treatment-limiting toxicity compared to ibrutinib. This superior safety profile should inform therapeutic decision-making and support the consideration of second-generation agents, particularly in patients with elevated cardiovascular risk, as a safer alternative to ibrutinib.

## Supplementary Material

oyag102_Supplementary_Data

## Data Availability

All data analyzed in this meta-analysis are derived from previously published studies, which are publicly available and have been cited in the references section. The extracted data are available from the corresponding author upon reasonable request.
