# Supplementary material for: Cardiac safety profiles of first-generation vs second-generation BTK inhibitors: a meta-analysis
Source: Oncologist. 2026 Mar 23;31(5):oyag102. doi: 10.1093/oncolo/oyag102 (PMC13105294; doi:10.1093/oncolo/oyag102)
Supplement: oyag102_Supplementary_Data [file oyag102_supplementary_data.docx]

**Cardiac Safety Profiles of First-Generation vs. Second-Generation BTK Inhibitors: A Meta-Analysis**

Ali Mushtaq^1^, Eman Nayaz Ahmed^2^, Roaa Aljumaa^2^, Abdel Rahman E’mar ^3^, Osamah Badwan ^3^, Omer Ashruf ^4^, Ahmad Elshaer^5^, Abdullah Shaik ^6^, Mohanad Baroudi ^1^, Rohit Moudgil ^3^, Moaath Khader Mustafa Ali^7*^

**Supplementary Methods**

**Supplemental Table S1: Risk of Bias Assessment Details**

A. Cochrane Risk of Bias 2 (RoB 2) Tool for Randomized Controlled Trials

| **Study** | **D1: Randomization Process** | **D2: Deviations from Interventions** | **D3: Missing Outcome Data** | **D4: Measurement of Outcome** | **D5: Selection of Reported Result** | **Overall Risk of Bias** |
| --- | --- | --- | --- | --- | --- | --- |
| ELEVATE-RR | Low | Low | Low | Low | Low | Low |
| ALPINE | Low | Low | Low | Some Concerns | Low | Some Concerns |
| ASPEN | Low | Low | Low | Low | Low | Low |

B. Newcastle-Ottawa Scale (NOS) for Observational Studies

| **Study** | **Selection (max 4 stars)** | **Comparability (max 2 stars)** | **Outcome (max 3 stars)** | **Total Score (max 9 stars)** | **Quality Assessment** |
| --- | --- | --- | --- | --- | --- |
| Roeker et al. | **** | ** | ** | 8 | Good |
| Qiao et al. | **** | * | ** | 7 | Good |
| Zhai et al. | ** | * | * | 4 | High Risk of Bias |

**Supplementary Figures: Forest Plots for Key Outcomes**

Forest plot comparing the risk of total cardiac events between first- and second-generation BTK inhibitors. Favors A represents a lower event rate with second-generation BTK inhibitors, while Favors B odds ratio (OR) compares first-generation (Group B) versus second-generation (Group A) BTK inhibitors. An OR < 1.0 indicates a lower event rate with second-generation BTK inhibitors (Favours A). Analysis was conducted using a random-effects model. Horizontal lines indicate the 95% confidence interval (CI). The diamond represents the pooled OR and its 95% CI.


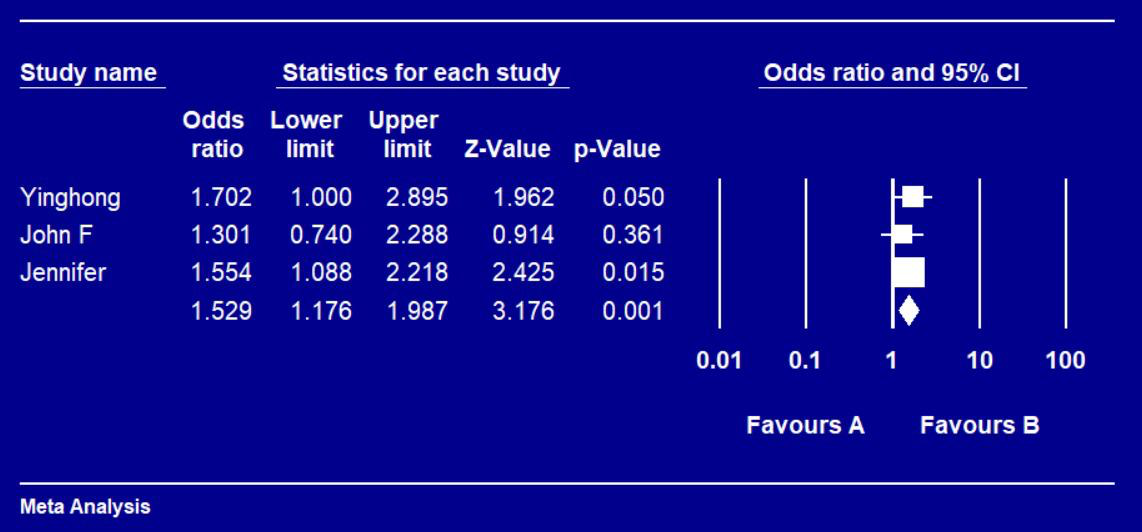


*Supplemental Figure S1: Risk of Total Cardiac Events*

*Forest plot comparing the risk of total cardiac events between first-generation (Group B) and second-generation (Group A) BTK inhibitors. An odds ratio (OR) < 1.0 favours second-generation BTK inhibitors. The analysis was conducted using a random-effects model. Horizontal lines indicate the 95% confidence interval (CI), and the diamond represents the pooled OR and its 95% CI.*


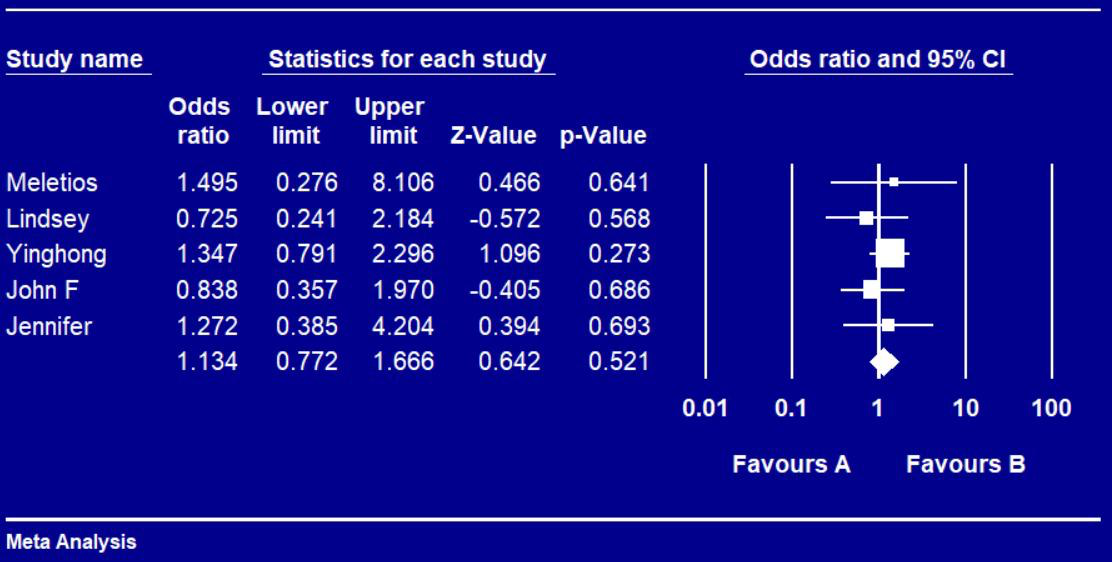


*Supplemental Figure S2: Risk of Heart Failure*

*Forest plot comparing the risk of heart failure between first-generation (Group B) and second-generation (Group A) BTK inhibitors. An odds ratio (OR) < 1.0 favours second-generation BTK inhibitors. The analysis was conducted using a random-effects model. Horizontal lines indicate the 95% confidence interval (CI), and the diamond represents the pooled OR and its 95% CI.*


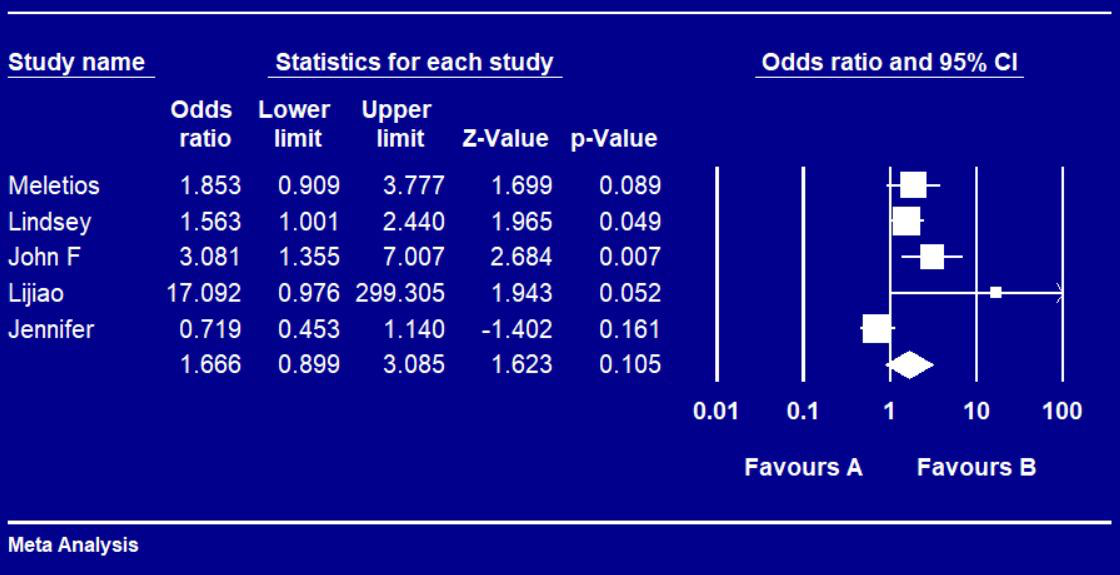


*Supplemental Figure S3: Risk of Hypertension*

*Forest plot comparing the risk of hypertension between first-generation (Group B) and second-generation (Group A) BTK inhibitors. An odds ratio (OR) < 1.0 favours second-generation BTK inhibitors. The analysis was conducted using a random-effects model. Horizontal lines indicate the 95% confidence interval (CI), and the diamond represents the pooled OR and its 95% CI.*


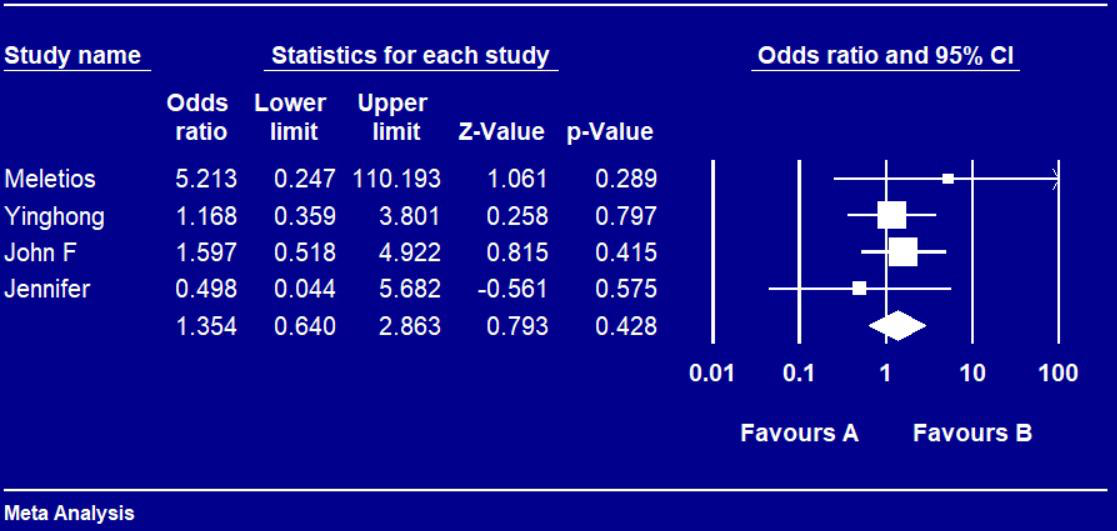


*Supplemental Figure S4: Risk of Ventricular Tachycardia*

Forest plot comparing the risk of ventricular tachycardia between first-generation (Group B) and second-generation (Group A) BTK inhibitors. An odds ratio (OR) < 1.0 favours second-generation BTK inhibitors. The analysis was conducted using a random-effects model. Horizontal lines indicate the 95% confidence interval (CI), and the diamond represents the pooled OR and its 95% CI.


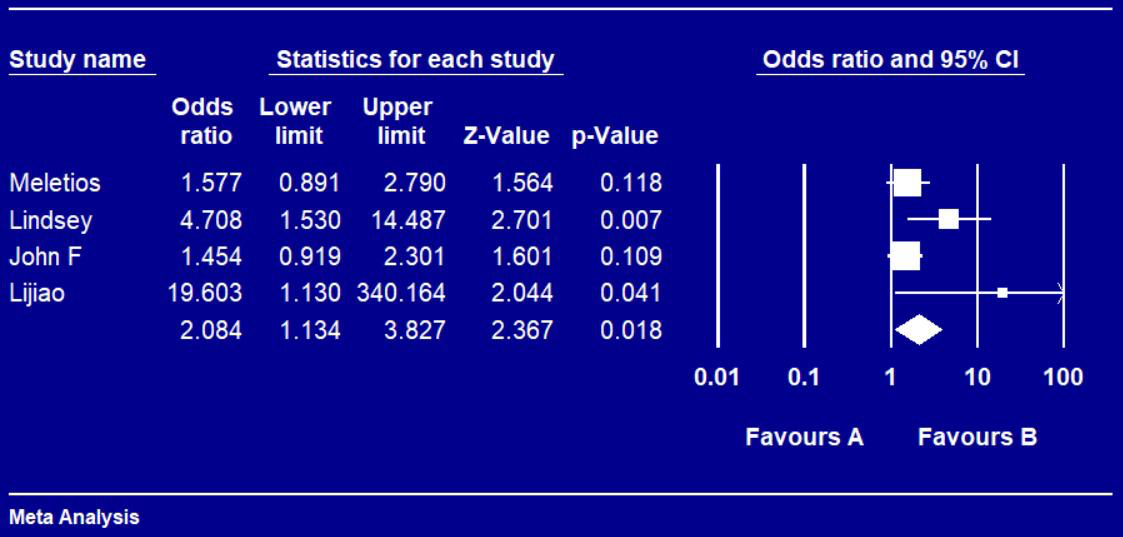


*Supplemental Figure S5: Risk of Bleeding Events*

*Forest plot comparing the risk of bleeding events between first-generation (Group B) and second-generation (Group A) BTK inhibitors. An odds ratio (OR) < 1.0 favours second-generation BTK inhibitors. The analysis was conducted using a random-effects model. Horizontal lines indicate the 95% confidence interval (CI), and the diamond represents the pooled OR and its 95% CI.*


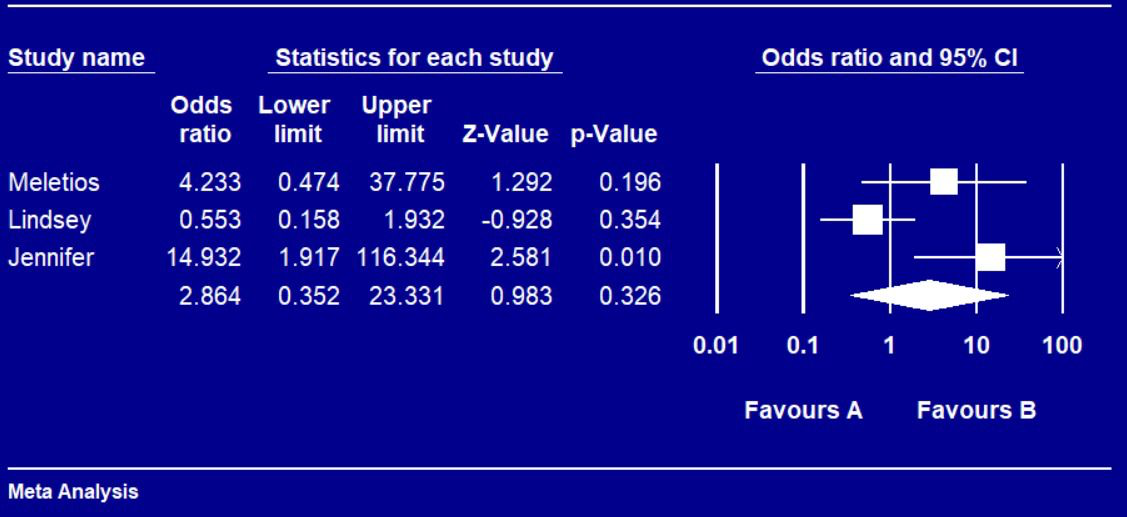


Supplemental Figure S6: Risk of Treatment Discontinuation due to Any Cardiac Event

Forest plot comparing the risk of treatment discontinuation due to any cardiac event between first-generation (Group B) and second-generation (Group A) BTK inhibitors. An odds ratio (OR) < 1.0 favours second-generation BTK inhibitors. The analysis was conducted using a random-effects model. Horizontal lines indicate the 95% confidence interval (CI), and the diamond represents the pooled OR and its 95% CI.


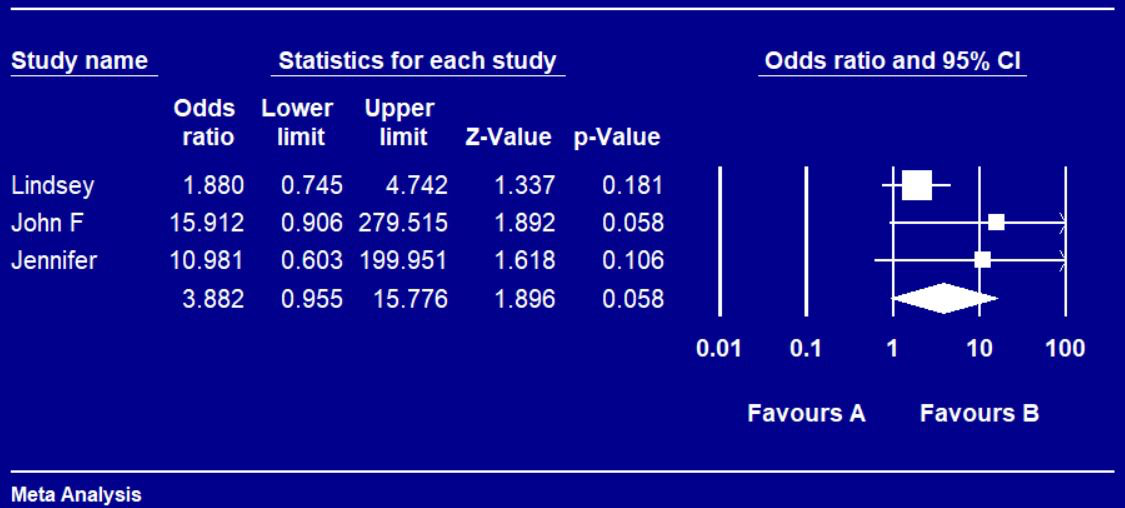


*Supplemental Figure S7: Risk of Treatment Discontinuation due to Atrial Fibrillation*

*Forest plot comparing the risk of treatment discontinuation due to atrial fibrillation between first-generation (Group B) and second-generation (Group A) BTK inhibitors. An odds ratio (OR) < 1.0 favours second-generation BTK inhibitors. The analysis was conducted using a random-effects model. Horizontal lines indicate the 95% confidence interval (CI), and the diamond represents the pooled OR and its 95% CI.*
